# Blunt cerebrovascular injury in rugby and other contact sports: case report and review of the literature

**DOI:** 10.1186/1749-7922-9-36

**Published:** 2014-05-04

**Authors:** Trajan A Cuellar, Lawrence Lottenberg, Frederick A Moore

**Affiliations:** 1 Division of Acute Care Surgery, Department of Surgery, University of Florida College of Medicine, Gainesville, FL, USA

## Abstract

Contact sports have long been a part of human existence. The two earliest recorded organized contact games, both of which still exist, include Royal Shrovetide Football played since the 12^th^ century in England and Caid played since 1308 AD in Ireland. Rugby is the premier contact sport played throughout the world with the very popular derivative American football being the premier contact sport of the North American continent.

American football in the USA has on average 1,205,037 players at the high school and collegiate level per year while rugby in the USA boasts a playing enrollment of 457,983 at all levels.

Recent media have highlighted injury in the context of competitive contact sports including their long-term sequelae such as chronic traumatic encephalopathy (CTE) that had previously been underappreciated. Blunt cerebrovascular injury (BCVI) has become a recognized injury pattern for trauma; however, a paucity of data regarding this injury can be found in the sports trauma literature.

We present a case of an international level scrum-half playing Rugby Union at club level for a local non-professional team, in which a player sustained a fatal BCVI followed by a discussion of the literature surrounding sport related BCVI.

## Case report

25 y/o male playing Rugby Union at scrum-half position was engaged in full contact training when he received a tackle. The exercise was a simple tackle drill, with two players at a standing start 10 meters apart. One player runs towards the other to initiate a tackle. The patient presented here received the tackle in an unremarkable fashion hitting the ground without loss of consciousness, then stood up briefly before collapsing. He was noted to be unresponsive and received CPR on scene and advanced medical intervention including intubation, placement of IV access and resuscitation before arriving as a trauma alert to UF Health Shands Level I Trauma Center in Gainesville, Florida.

On arrival in the trauma bay his vitals were GCS 3 T, HR 60s with a bradycardic episode to 30s that was short lived, and SBP 97 with on-going fluid resuscitation. ATLS primary and secondary surveys were completed along with laboratory investigations. A central line and arterial line were placed along and the patient received a CT head 24 minutes after ambulance arrival. This revealed a diffuse SAH in a non-traumatic pattern. The imaging protocol was then altered in the CT scanner to include a CT angiogram of the head/neck that confirmed a right-sided internal carotid dissection with occlusion of the right ICA at the junction of the right cavernous sinus and supraclinoid ICAs. Mannitol and 3% saline were administered and a ventriculostomy was placed. CSF fluid was noted to be grossly bloody. Maximal medical therapy continued overnight with repeat CT head revealing right ICA dissection, large volume SAH extending into high convexity sulci bilaterally with early central incisural herniation, right MCA and ACA stroke, and right ACA distribution cytotoxic edema.

At 24 hrs following admission, the patient was noted to have new left sided pupillary dilatation with ICPs that remained in 70s despite maximal medical therapy.

**Figure 1:**
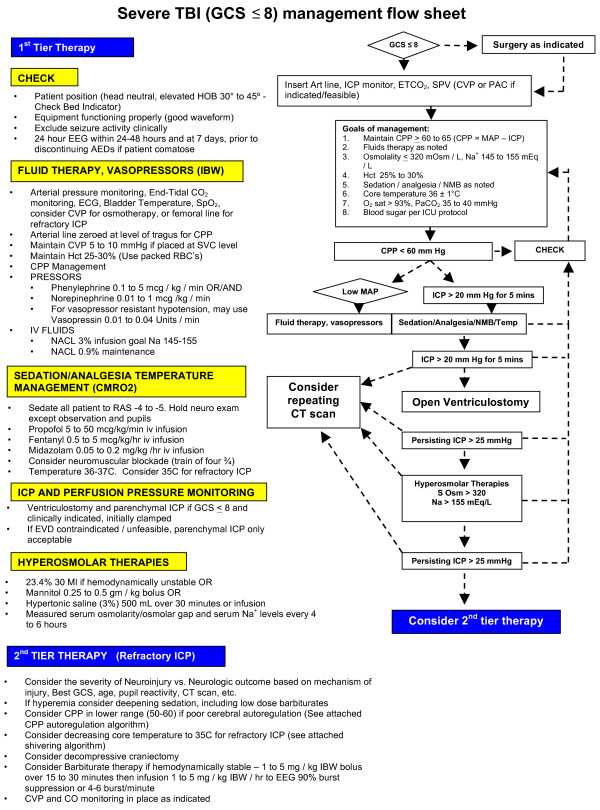
CTA brain transverse image demonstrating occlusion of right internal carotid artery.

**Figure 2:**
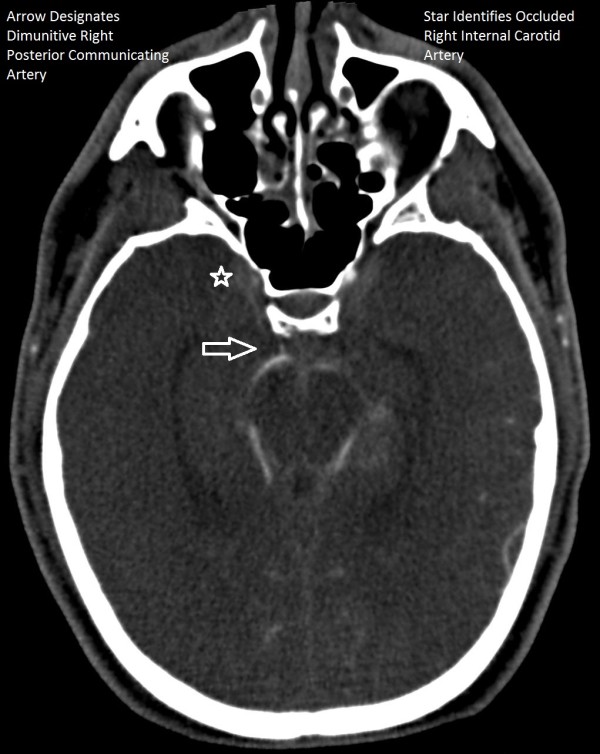
CTA brain coronal image demonstrating diminutive right posterior communicating artery.

His clinical condition continued to deteriorate and he was pronounced brain dead ~36 hrs after admission with the family electing to withdraw care upon arrival of other family members. Two CT Angiograms demonstrating his Grade IV BCVI injury are provided below (Figures 1 and 2).

**Figure 3:**
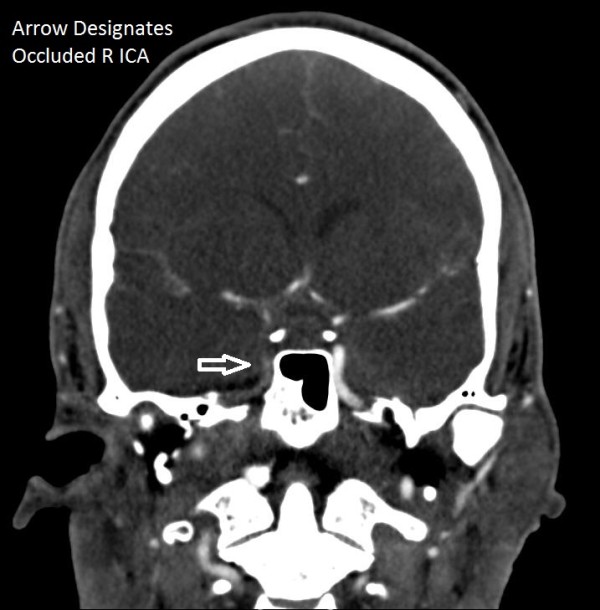
University of Florida severe brain injury algorithm.

A list of Denver BCVI screening criteria is listed below:

The Denver criteria for screening for BCVI in context of trauma includes any cervical fracture, unexplained neurological deficit, basal cranial fracture into the carotid canal, Le Fort 2 or 3 fracture, cervical hematoma, cervical bruit, ischemic stroke, or head injury with GCS <6. Below is the University of Florida Severe Brain Injury Protocol which was followed during the treatment of this patient (Figure 3).

## Discussion

Thus far, there exist a total of 3 case reports of cerebrovascular accident associated with blunt trauma in Rugby.

The first is a 15 year old playing hooker (middle front row in the scrum) with a trauma associated CVA that presented with primarily sensory symptoms that included neck pain and paresthesia of right arm and leg [1]. He was removed from the game and did not return to play. He developed additional symptoms the following day including dizziness and blurred vision with ongoing right upper extremity paraesthesia. MR imaging revealed an infarct in the anterior limb of the internal capsule and the head of the caudate nucleus. A diagnosis of carotid dissection was made as a source without angiography based on history and distribution of infarct the patient. This was treated conservatively without anticoagulation or antiplatelet therapy with near full resolution of his symptoms with residual numbness of the hand at follow up 4 weeks later.

The second case is a 31 year old who sustained a ‘fierce hand off’ to the right neck while playing but continued to play without neurological signs or symptoms [2]. He then presented 2 weeks later to the ED with right neck swelling and pain with shortness of breath and a diagnosis of ruptured pseudoaneurysm of the common carotid was made with subsequent open surgical intervention. He had a presented to a general practitioner one week post injury and received antibiotic therapy for a swollen gland in the neck. Interestingly he had no neurological symptoms or signs as part of his presentations.

The third is a 19 year old rugby player who sustained a posterior sternoclavicular dislocation that required he retire from the game [3]. He had no neurological signs or symptoms, only pain associated with the injury. He then presented 3 weeks post injury with dizziness and collapse on the rugby pitch, which was diagnosed as secondary to two vascular injuries one of the right proximal subclavian artery and the other of the innominate artery. He received surgical intervention including a median sternotomy, and at 1 year had residual neurological deficit of left UE and LE.

Additional case reports of BCVI in include a series of 5 cases that include one sport-related BCVI. This case was an 18 year old male struck in the neck while playing basketball and was ‘moderately dazed’ but reentered the game and continued to play [4]. He then presented 2 weeks post injury with acute hemiplegia and was diagnosed with carotid dissection and underwent surgical intervention but developed and large left sided hemispheric infarct and expired 5 days post admission. This case series included trauma patients and highlighted the delayed nature of presentations of BCVI with new neurological deficits ascribed to the injuries occurring as late as 6 months post injury [4]. Similarly, a case series from Mayo Clinic of 18 patients 3 of which were sports related injuries, also noted a delay in presentation from 30 minutes to 10 years post injury [5]. Within the pediatric literature there are individual case reports including a report of 3 American football players 17, 15, and 14 years of age who sustained cerebellar infarct, left pontine stroke, and left middle cerebral infarct respectively [6]. These players all had neurological findings and also presence of one or some of the following prothrombotic mutations: methylene tetrahydrofolate reductase gene variant C677T and A1298C, PAI 1-4G, prothrombin 20210.

Additionally, there is a report of a 15 year old who developed symptoms during a game of American football without obvious trauma and presented to hospital with a progressive neurological deficit ascribed to a left ICA dissection with hemispheric infarct and an ultimately fatal course 4 days following admission [7]. It is unclear from the case report whether or not he was playing.

A review of 18 cases of sport-related BCVI (not including Rugby) were related to a wide range of activities including cycling, football, French boxing, Hockey, In-line Skating, Scuba diving, Skiing, Softball, Taekwondo, Weight lifting, and Wintersports [8]. Pathophysiology was presumed to be due to a crush injury to the carotid with disruption to the intima in 62% of patients with a subintimal dissection with internal carotid dissections carrying a more severe course and worse long term outcome.

In a recent broad overview of BCVI etiology is thought to be stretch of the common carotid artery over C3-5 during extreme neck extension [9]. The strokes that arise from these injuries are thought to be either embolic from dislodged clot from a focal site of intimal disruption or from dissection causing vessel occlusion or sufficient narrowing to result in cerebral infarct. Anatomic variation in the Circle of Willis, incomplete in 80% of the population, contributes to the severity of carotid occlusion by functionally making the internal carotid artery an end artery rather a collateralized artery. This fact is further corroborated from recent vascular surgery literature regarding 2 or more obstructions or agenesis within the Circle of Willis with inability to tolerate carotid cross clamping [10].

Regarding our case the patient received a traumatic tackle while playing at scrum-half position (back) in a training scenario. Such contact is inevitable in the course of training and play, however, epidemiological studies in Rugby do exist and have been used to identify risk factors that are associated with injury and propose effective strategies to reduce the number of injuries. Stricter adherence to rehabilitation plans, reduction in the amount of foul play, and improvement in the quality of the pitch specifically with regards to hardness were identified as risk factors for injury [11]. A recent review regarding injury in Rugby Union states that there is no difference in injury rate between forwards and backs with the majority of injuries being sustained in a tackle or scrum [12]. Indeed the majority of injuries occur not during practice but in a competitive match at a ratio of 36:1 and usually to the backs in the context of an open field tackle during which time there is more high energy transfer than other portions of the game. Catastrophic spinal injuries were noted to be relatively rare at 1 per 10,000 players per season and again normally sustained in the context of the scrum or tackle in open field play.

American football a sport with similar goals to rugby has been studied in greater detail, but still lacking in data resolution to identify BCVI as a sub-cohort of injury pattern. In a review article in 2013 Boden et al [13] noted out of 164 traumatic American football fatalities only one death from vascular injury in conjunction with cervical fracture was found but there were 5 deaths due to brain injury without ascribable cause. It is conceivable that BCVI may have been involved in these deaths.

Additionally, a comparative study between American Football and Rugby has demonstrated differences in volume of injury (3 times higher in Rugby compared to American football) [14]. Also, differences in the injury pattern include a higher rate of neck injuries in Rugby 1.02 compared to 6.02 per 1000 player games [12]. The nature of neck injuries is also different with American Football players experiencing traumatic distraction of the brachial plexus with upper extremity neurological symptoms frequently called a ‘stinger’, which was shown to occur up to 50-65% of collegiate level American Football players [15]. Interestingly this injury pattern appears absent in Rugby.

It may be in Rugby the majority of neurological symptomatology of the upper extremity are the result of manifestations of vascular injury with neurological sequelae rather than neurological injury. For the player with symptoms this means a more focused assessment of vascular structures may be warranted upon identification of neurological signs or symptoms.

BCVI in the trauma literature is a treatable disease with delays having serious consequences [16-19]. In the trauma literature a review of 147, BCVI cases highlighted the positive effect of treatment with stroke found in 25.8% of untreated patients and 3.9% of treated patients [18]. Indeed in the trauma population 30% of undiagnosed BCVI will go on to produce strokes [16]. Usage of CTA to diagnose and guide therapy for BCVI in trauma population has had encouraging results with great reductions in delayed stroke rate from 67% to 0% and mortality reduction from 38% to 10% [19]. The single best predictor of positive screening for BCVI was symptomatic presentation [20]. Protocols have been published regarding specific treatment of injury by grade which may guide treatment in low-energy sport injuries [21].

At the higher level of the game a review of Elite Irish Rugby Players reveal under-reporting of blunt concussive injury by as much as 41% [22]. This underreporting phenomenon is not restricted to Rugby with only moderate reliability of reporting concussive events in former professional American Football players [23].

## Conclusion

Rugby Union is a high energy contact sport that is widely played in the USA with over 2,800 active clubs and over 450,000 players. Blunt cerebrovascular injuries associated with rugby are rare events but can have subtle presentations and ultimately catastrophic outcomes. No data exists regarding the rate of BCVI in contact sports, their grade, or their chance of progression to stroke over time. What is known about BCVI in Rugby or other contact sports is that it is documented to exist mainly in an anecdotal form, which may over time form a cohort of data. BCVI outside sports within the trauma literature is noted to be progressive with 29% of injuries deteriorating over time and 30% producing stroke over time. Additionally, the time to stroke may not be immediate with delays in presentation being common in the sports literature. Treatment is effective in reducing stroke rate and mortality.

As the Rugby World Cup of 2015 approaches with no data regarding epidemiological studies of BCVI in Rugby; it is worth noting this injury can have devastating consequences and further study is needed to delineate its nature and to ensure appropriate screening of those players who suffer injury with neurological signs. Additionally, those players who require treatment and are identified as having neurological symptoms may benefit from enhanced symptom/sign screening to elucidate the nature of these injuries and gather data to help delineate strategies to predict and prevent a catastrophic outcome with timely medical intervention. Inclusion of neurological screening questions as part of an assessment for BCVI by trained medical personnel with application of CT Angiography in players undergoing CT imaging for TBI or maxillofacial injury should be considered. Most important, robust documentation of injuries including those with neurological signs/symptoms should be implemented to provide data on injury patterns in Rugby Union with leadership provided by the International Rugby Board [24].

## Competing interests

The authors declare that they have no competing interests.

## Authors’ contributions

All authors read and approved the final manuscript.
